# Security challenges by AI-assisted protein design: The ability to design proteins in silico could pose a new threat for biosecurity and biosafety

**DOI:** 10.1038/s44319-024-00124-7

**Published:** 2024-03-26

**Authors:** Philip Hunter

**Affiliations:** Freelance Journalist, London, UK

**Keywords:** Biotechnology & Synthetic Biology, Computational Biology, Science Policy & Publishing

## Abstract

Scientists and security experts are concerned that the increasing power of AI-assisted protein design and synthesis could be abused by various actors for terrorist or criminal purposes.

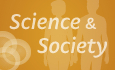

The COVID pandemic has, among other things, intensified the debate about biosecurity and biosafety, driven by concerns not only about naturally emerging diseases but also accidental releases of pathogens and nefarious uses of biotechnology. It has, for instance, inspired the Pathogens Project by the Bulletin of the Atomic Scientists, which published a report on alleviating biological threats posed by technical advances earlier this year (https://thebulletin.org/pathogens-project/report-2024/?utm_source=Website&utm_medium=SitewideBanner&utm_campaign=SitwideBanner_02282023&utm_content=DisruptiveTechnologiesBio_PathogensProjectReport_02282023).

One of those concerns regards the rapid progress both in protein design and synthesis, and potential nefarious uses of these technologies. This has led to a notable proposal in January 2024 for collecting and storing the underlying DNA sequence data of artificial proteins for screening purposes by David Baker and George Church, eminent researchers respectively in computational protein design and DNA synthesis (Baker and Church, 2024). Their concerns were especially raised by the rapid increase in power and accuracy of computational protein design resulting from incorporation of AI techniques and especially Generative AI (Gen AI).

The real or potential threats being addressed by Baker and Church come under three headings, according to Nicole Wheeler, whose team develops computational screening tools for identifying DNA from emerging biological threats at the University of Birmingham in the UK. “The first is a potential improvement in the access to DNA encoding known biological hazards,” she said, even if DNA synthesis orders are screened for toxin or pathogen-related sequences. “This screening relies on sequence similarity to known hazards and could be undermined by the ability to create novel sequences with similar functions to known proteins but with undetectable sequence homology.”

“This screening relies on sequence similarity to known hazards and could be undermined by the ability to create novel sequences with similar functions to known proteins but with undetectable sequence homology.”

The second concern lies in the application of protein design tools to optimize existing hazards in various ways. This could be to make them more dangerous, by increasing the toxicity of known toxins, or improving viruses’ ability to escape existing immunity. It could also make viruses more transmissible or lethal. Furthermore, complementary efforts to apply machine learning for prediction of host range, transmissibility and virulence of pathogens could make it easier to perform such hazard optimization.

The second concern lies in the application of protein design tools to optimize existing hazards in various ways.

The third risk category is the growing ability to design completely novel toxins, which could in principle target a human protein immune to naturally occurring toxins. “The better we understand the vulnerabilities of human, animal and plant systems, the greater the attack surface we create for malicious actors,” Wheeler warned. She acknowledged though that the second and third concern categories are more speculative at present, but indicated they also pose the greater risk and are not properly addressed by existing measures in place for combating biological threats.

The third risk category is the growing ability to design completely novel toxins, which could in principle target a human protein immune to naturally occurring toxins.

## Protection against synthetic biological harms

Baker and Church set out to establish a practical barrier against harmful biomolecules in all these categories, whether created accidentally or intentionally. They pointed out that the biological complexity involved makes it highly unlikely that a dangerous agent capable of effective manufacture and dissemination could be created in a single attempt, even with the help of Gen AI.

As a result, the collection and storage of protein DNA sequences set out in their proposal should be able to trace nascent threats back to their origins. “Besides providing an audit trail, awareness that all synthesized sequences are being recorded may deter bad actors,” Baker and Church wrote. “Screening and logging practices should be standardized, practiced internationally, and extended to benchtop nucleic acid synthesizers.”

… the biological complexity involved makes it highly unlikely that a dangerous agent capable of effective manufacture and dissemination could be created in a single attempt, even with the help of Gen AI.

When approached for comment, Baker referred to Ian Haydon from the Institute for Protein Design at the University of Washington where he is Head of Lab. “It’s important to remember that no computationally designed biomolecule can possibly cause harm unless it is physically produced,” Haydon said. “That’s why screening and logging all manufactured DNA sequences is so important. Securing this ‘digital-to-physical divide’ is the most practical way to catch potential mistakes and detect and deter bad actors, all while preserving innovation. For this to work, we’ll need standardized protocols and international cooperation.”

## The potential of AI-assisted protein design

Baker and Church came together because they cover protein design and the DNA synthesis between them. Baker has recently worked on application of deep-learning technologies, such as RFdiffusion and ProteinMPNN, to generate biomolecules with new functions. Church was a pioneer of DNA sequencing and synthesis at Harvard University. The accelerated progress in de novo protein design and potential for further advances resulting from the relatively sudden and unexpected advance of Gen AI, in the absence of clearly agreed and applied international protocols, prompted them to devise their scheme.

Popularized by Open AI’s Chat GPT now on its fourth version, Gen AI harnesses ever increasing computing power to build on existing AI techniques founded on multilayer neural network-based machine learning, with ever greater predictive power in many fields. It could almost be called generalized AI, but also appears more creative because it can predict scenarios or outcomes, consistent with the data sets upon which the models are trained.

Such outcomes can be images or video generated from underlying text as one example, or they can be protein structures conforming to a preset target conformation. Indeed, Google’s DeepMind AlphaFold, which was based on AI techniques to predict 3D protein structures from the underlying sequence, culminated in the release of predicted structures for most catalogued proteins in conjunction with the European Bioinformatics Institute (EMBL-EBI) (AlphaFold reveals the structure of the protein universe - Google DeepMind).

The concerns addressed by the Baker/Church proposal are almost the opposite process of working backwards ultimately to a DNA sequence that can be applied in transgenic organisms to generate the desired protein at scale. As they noted, computational protein design methods have started to deliver some notable therapeutic successes, such as the Covid-19 vaccine SKYCovione, the first drug developed this way to be approved for use (COVID-19 vaccine with IPD nanoparticles wins full approval abroad - Institute for Protein Design (uw.edu)).

## Possible drawbacks and limitations

The Baker/Church proposal begs several questions though. One is whether it will gain sufficient traction, especially outside the USA, to have a chance of meeting its ambition of curtailing the development of dangerous products. Then, as Wheeler noted, there is the question of how well it will deter hostile actions by states, terrorist groups or deranged scientists in academic or industry labs, even if it may be successful at curtailing accidental releases. “This type of screening is unlikely to deter state actors, as they would be capable of producing their own DNA without having to go through providers who screen,” she said. “Safeguards on the design tools themselves would be a better intervention point to deter state actors, such as controlled, monitored, and logged access to AI protein design capabilities.” She pointed out that states were already building their own AI tools, China being a leading player on this front.

Another issue is the extent to which the Baker/Church proposal builds on existing protections, and which elements of it have already been implemented. In fact, DNA synthesis has been regulated since 2004 by a proposal from the International Gene Synthesis Consortium (IGSC), which has been widely adopted among academia, as well as by many biotech and pharma firms, on a voluntary basis. Under this protocol, requests to academic, private and government institutions for DNA sequences are screened for homology to pathogen components from a consensus list.

…DNA synthesis has been regulated since 2004 by a proposal from the International Gene Synthesis Consortium (IGSC), which has been widely adopted among academia, as well as by many biotech and pharma firms…

However, as Baker and Church argue, current screening has become outdated by the increasing ability to generate de novo proteins, which, by definition, are not guaranteed to be homologous to existing natural proteins, and often will not be. There is a need therefore to scrutinize newly synthesized sequences and add them to protein databases, using a combination of encryption and strong authentication to protect them. Baker and Church propose that screening checks in future should be integrated with the synthesis process itself. This would require that each new genetic sequence is authenticated through a cryptographic process before it can be synthesized by a given machine.

## The global picture

Among those cautiously optimistic but clear about the limitations of such a protocol is Tom Inglesby, Director of the Johns Hopkins Centre for Health Security in Baltimore, USA. “Establishing legal requirements for screening genome synthesis orders is a critical and important step that governments can take to reduce risks related to the design and synthesis of harmful manufactured genetic materials,” he commented. “If a strong screening system is required by a country’s law, it would help lower the chances that harmful molecules that are designed in silico are translated into physical molecules. But it’s also important to acknowledge that this kind of screening, while very important, is not an absolute barrier to harmful manufactured genetic materials being made from in silico designs. If in silico designs for harmful molecules are published openly online, they are then available globally from that point forward.”

Ingleby noted that at present there were few if any compulsory screening requirements in any country. “Even if there were, they alone would not prevent determined actors with scientific expertise and the right tools from translating in silico designs into actual molecules,” he added.

A similar point was made by Wheeler. “If governments themselves were determined to use genome synthesis as a means of doing harm—recognizing that intentional misuse would be a violation of the Biological Weapons Convention—then screening requirements would not likely stop them,” she said. But, she added that most states, including China, are themselves interested in preventing high-consequence misuse of biology. “So the goal here should be that all countries set up screening requirements to create high barriers to rogue scientists or non-state actors synthesizing molecules that could do high harm.”

As Wheeler hinted, the risks posed by rogue scientists in labs acting on their own will probably exceed that from state actors or organized terrorist groups. There is the risk though of such lone wolves being planted by some malevolent agency, which could conceivably be a state or terrorist group.

As Wheeler pointed out, while universal logging of manufactured DNA, as proposed by Baker and Church, would be a simple measure that could deter such bad actors, sophisticated screening is also required to snuff out dangerous proteins at the in silico stage before they reach manufacture. “Enhanced screening is a more technically challenging task which should be receiving attention and funding, as it presents a key bottleneck that could potentially prevent dangerous designs from becoming a physical reality,” Wheeler explained. “A major barrier to the success of this approach at the moment is the computational cost of next-generation screening to detect AI-generated hazards. More powerful tools to detect dangerous sequences, such as structure-based homology prediction, are computationally intensive to run, and the DNA synthesis industry must regularly screen an enormous volume of orders. Research into minimizing this computational cost would make this venture more achievable.”

However, Margaret Kosal, Director of the Military Fellows Programs at the Sam Nunn School of International Affairs at Georgia Institute of Technology in Atlanta, USA, who specializes in biosecurity, is more sanguine at least about the impact of AI on biosecurity generally. She cited a 2022 paper describing how AI methods could be applied to design toxic molecules (Urbina et al, 2022*)*. “Like earlier approaches to drug design via combinatorial chemistry, the authors importantly note that their AI models were ‘originally created for use in avoiding toxicity, enabling us to better virtually screen molecules (for pharmaceutical and consumer product applications) before ultimately confirming their toxicity through in vitro testing.’ The driving motivation of the research and development of capabilities is for beneficial purposes. This is important to highlight,” Kosal explained.

… sophisticated screening is also required to snuff out dangerous proteins at the in silico stage before they reach manufacture.

“It’s also important to recognize—and it often gets a lot less attention—that there are opportunities for AI/ML to contribute to non-proliferation efforts, including in context of protein synthesis, through the development of AI/ML capabilities to detect, deter and limit proliferation,” Kosal added. “Far-future artificial intelligence may be able to track proliferator progress, anticipate nuclear decision points, and design new arms reduction frameworks. That’s important potential applications that should be highlighted as well.”

This leads to the important point that risks posed by de novo protein design can only be properly understood in the context of motivations and intensions on the part of the actors involved, and not just on the technological front. Geopolitics and motivations need to be factored into the deliberations, as Kosal pointed out. “What is desperately needed more in the discussion is consideration of motivation—why would a state pursue a technologically-enabled bioweapon? Rather than nuclear or conventional weapons? What advantages would it convey? What are the strategic, operational, or tactical aims that would drive a state? For example, is a state trying to overcome a specific countermeasure, such as vaccine or other therapeutic, that a competitor relies on? Is there are path that they can execute?”

Kosal also underlined the complexities involved, which tend to encourage greater focus on nuclear and chemical Weapons of Mass Destruction. “While such AI tools may lower barriers to entry for an extremely motivated actor, like in a sophisticated state-based program, to design highly toxic compounds in silico, using the databases of hypothetical compounds to actually create the chemicals, never mind weaponize them, still also requires sophisticated chemistry and engineering expertise and materials to synthesize the candidate compounds and to handle them safely until deployed. This is a difficult task for known chemicals, but one which becomes more difficult for novel chemicals whose properties have not yet been characterized or studied.”

Kosal agreed that AI/ML-enabled in silico drug design holds great potential promise for biomedicine, biomaterials and other beneficial applications, but these legitimate applications are some way from realization. “Anyone who has done experimental synthetic chemistry, whether organic, inorganic or solid-state, knows that there is huge gap between coming up with a predicted molecular structure with desirable properties and firstly synthesizing it, secondly getting an actual crystal structure, usually via X-ray crystal diffraction rather than powder diffraction, and thirdly actually characterizing its properties, such as reactivity, stability, and catalysis.”

## Globally accepted security policies

Kosal shared the general approval of the Baker and Church proposal, not least because their call is more likely to be heard given their prestige. But she wondered how the proposed screening will be implemented in practice. “In the context of the US Executive Order (governing AI risks and introduced by President Biden in late 2023), AI regulations are likely to be tied to federal funding, that is from the NIH, NSF, etc, and thus will largely affect basic research work done at universities,” she said. “That’s likely not the source of most risk, especially in context accidents. For example, commercially available synthesis by companies in nation-states with lax regulation or limited enforcement would not be affected.”

Kosal added that the risks existed before the advent of AI, which has merely enhanced them and drawn attention to them. “Something good is that perhaps the attention on AI can be leveraged to drive more effective implementation and enforcement of policies that were needed before all the attention on AI.”

She made three other critical points though. First is that efforts to curtail the threats should not be too US-centric, even if the USA has taken a lead with the Baker/Church proposals. “Other approaches that don’t get as much attention and are focused outside the US are the type of activities that have been funded by the US State Department’s State’s Cooperative Threat Reduction Program,” Kosal added. “It’s subtler—but ultimately about building relationships, security, and sharing best practices that are implementable outside the US. There has been a series of these workshops. […] One might call it an attempt to inoculate against misuse of the life sciences and biotechnology pro-actively.”

Secondly, Kosal pointed out that strong support for traditional approaches, such as the Biological Weapons Convention (BWC) and the UN’s Implementation Support Unit (ISU), were crucial in reducing misuse of the life sciences and biotechnology.

Finally, she underlined the need to avoid being too heavy-handed and throwing out the baby with the bathwater, as the old German proverb goes. She cited the US National Security Presidential Memorandum 33 (NSPM-33) on research security, which she suggested may be pushing US universities to a point where the principles are becoming so onerous they are potentially counter-productive.

“Often researchers and faculty have little or no input on the design and implementation of programs at individual universities, which can lead to bad or unimplementable policies,” she explained. “Everyone understands that research security is important. But it’s clear that some of the implementers don’t understand research. Adding more restrictions related to protein synthesis on university researchers is adding another link in an already very long chain.” It increases the risk of deterring or discouraging potentially beneficial research, especially by startups and spinoffs seeking to concentrate their resources on the development in hand. On this last front, initiatives such as the US Department of Defense’s Biomanufacturing initiative (BioMADE) come into play by testing and shrink-wrapping implementable principles that can then also be exported to other countries.

“Everyone understands that research security is important. But it’s clear that some of the implementers don’t understand research.”

There is a lot happening then on the regulatory front around protein synthesis and application of AI, although at present much of it is in the USA. More attention is needed in other countries and at least the Baker/Church proposals have raised awareness.

### Supplementary information

Peer Review File
