# Supplementary material for: Security challenges by AI-assisted protein design: The ability to design proteins in silico could pose a new threat for biosecurity and biosafety
Source: EMBO Rep. 2024 Mar 26;25(5):2168–71. doi: 10.1038/s44319-024-00124-7 (PMC11094011; doi:10.1038/s44319-024-00124-7)
Supplement: Supplementary file 1 — Peer Review File [file 44319_2024_124_MOESM1_ESM.pdf]

EMBO reports

Security challenges by AI-assisted protein design

Philip Hunter

Corresponding author(s): Philip Hunter (ph@philiphunter.com)

Review Timeline:

Submission Date:	14th Mar 24
Accepted:	15th Mar 24

Editor: Holger Breithaupt

Transaction Report: This article was editorially reviewed.
